# Effects of antibiofilm compounds on the cellular and bacterial colonization of polimeric surfaces: a step towards biofunctionalization of implantable devices

**DOI:** 10.1186/1753-6561-8-S4-P71

**Published:** 2014-10-01

**Authors:** Armando Rodrigues Lopes Pereira Neto, Lya Piaia, Camila Quinetti Paes, Aguedo Aragones, Ricardo de Souza Magini, Luismar Marques Porto, Ulisses Alves Pereira, Luiz Claudio de Almeida Barbosa, Andrea de Lima Pimenta

**Affiliations:** 1 Department of Chemical Engineering and Food Engineering, Federal University of Santa Catarina (UFSC), Integrated Technologies Laboratory - Florianópolis, Santa Catarina, Brazil; 2 Department of Odontology, Federal University of Santa Catarina (UFSC), Dental Implants Teaching and Research Center - Florianópolis, Santa Catarina, Brazil; 3 Department of Chemistry, Federal University of Viçosa - Viçosa, Minas Gerais, Brazil

## Background

Periodontal and peri-implant diseases are infection pathologies related to the pathogenicity of the microorganisms involved [1]. The worldwide practice of indiscriminate and continuous use of antibiotics for the control and prophylaxis of bacterial pathogens has led to the development of bacterial resistance to most available antimicrobials [2]. Concurrently, the rapid increase in bacterial resistance to currently available antimicrobial drugs has led researchers to search for new sources of molecules active against bacterial pathogens, outlining a new generation of anti-infective drug development. A promising approach for the development of a new generation of antimicrobial drugs has arisen from the studies of host-pathogen interactions, which prompted a shift of drug targets from bacterial survival to pathogenicity control. Examples of potential non-conventional targets for microbial control are molecules and receptors involved in bacterial adherence to biotic and abiotic surfaces as well as signal systems controlling bacterial group behaviour of populations organised in biofilms, such as *quorum sensing *(QS) [3]. In contrast to conventional antibiotics, antimicrobial drugs directed against such unconventional targets do not jeopardise bacterial survival, imposing a low selection pressure and thus avoiding the development of resistance [4].

## Methods

Here we analysed the effects of novel synthetic lactams, compounds derived from furanones which display recognized antibiofilm activity. Three synthetic lactams were tested against biofilm of *Enterococcus faecalis *grown over an implantable polymeric material (PLGA-HA). Inhibition assays of adhesion and biofilm formation, and SEM observation were used to quantify and qualify *E. faecalis' *biofilm formation. To assay for biofilm inhibition, bacterial strains were grown statically for 20 h at 37°C in TSB. Polystyrene 96-well microtitre plates were inoculated with 100 μL/well of bacterial suspension previously diluted to 5 × 10^8 ^CFU/mL in TSB supplemented with 4% sucrose (w/v) and 3.5% (v/v) dimethyl sulphoxide (DMSO). Compounds to be tested for antibiofilm activity were prepared at a concentration of 175 μg/ml, in the same media as before, and added to the test wells. Biofilm inhibition capacity was assessed by CFU counting after bacterial disaggregation from the surfaces and microscopic analysis of the polymers. Cytotoxicity of the lactams against human fibroblasts and keratinocytes was tested, at four different concentrations (43,75; 87,5; 131,25 and 175 μg/ml), by MTT assay [5].

## Results and conclusions

All compounds showed biofilm inhibition rates higher than 99%, as accessed by CFU counting and in agreement with images obtained by SEM. At the MIC (minimum inhibitory concentration) human cells viability indexes of all compounds were higher than those recommended for approval in clinical use. Results presented here indicate that lactams have excellent potential for use in the treatment of periodontal disease and in association with PLGA-HA can be used in the prevention of peri-implantitis disease.

## References

[B1] Socransky SS Haffajee AD The bacterial etiology of destructive periodontal disease: current concepts J Periodontol 1992 63 Suppl 3 322 331 29539682 10.1902/jop.1992.63.4s.322

[B2] Richards JJ Melander C Controlling bacterial biofilms Chembiochem 2009 10 2287 2294 10.1002/cbic.200900317 19681090

[B3] Chen G Swem LR Swem DL Stauff DL O'Loughlin CT Jeffrey PD Bassler BL Hughson FM A strategy for antagonizing quorum sensing Mol Cell 2011 42 199 209 10.1016/j.molcel.2011.04.003 21504831 PMC3092643

[B4] Kolter R Biofilms in lab and nature: a molecular geneticist's voyage to microbialecology Int Microbiol 2010 13 1 7 20890834 10.2436/20.1501.01.105

[B5] Sieuwerts AM Klijn JG Peters HA Foekens JA The MTT tetrazolium salt assay scrutinized: how to use this assay reliably to measure metabolic activity of cell cultures in vitro for the assessment of growth characteristics, IC50-values and cell survival Eur J Clin Chem Clin Biochem 1995 33 813 823 8620058 10.1515/cclm.1995.33.11.813

